# Paclitaxel-coated balloon treatment is associated with sympathetic coronary denervation in a porcine model

**DOI:** 10.1038/s41598-026-54572-3

**Published:** 2026-06-04

**Authors:** Mert Tokcan, Stephanie Bettink, Ole Gemeinhardt, Saarraaken Kulenthiran, Michael Böhm, Felix Mahfoud, Bruno Scheller

**Affiliations:** 1 Klinik für Innere Medizin III – Kardiologie, Angiologie und Internistische Intensivmedizin, Saarland University Medical Center, Saarland University, Kirrberger Straße 100, 66421 Homburg, Germany; 2 Clinical and Experimental Interventional Cardiology, Faculty of Medicine, Saarland University, Homburg, Germany; 3 Department of Radiology, Charité - Universitätsmedizin Berlin, Corporate Member of Freie Universität Berlin, Humboldt-Universität Zu Berlin, Berlin, Germany; 4 Department of Cardiology, University Heart Center, University Hospital Basel, Basel, Switzerland; 5 Cardiovascular Research Institute Basel (CRIB), University Heart Center, University Hospital Basel, Basel, Switzerland; 6 Department for Biomedical Engineering, University of Basel, Basel, Switzerland; 7 HOMICAREM (HOMburg Institute of CArdioREnalMetabolic Medicine), Medical Faculty, Saarland University, Homburg, Germany

**Keywords:** DCB, Drug-coated balloon, Coronary innervation, Late lumen enlargement, Periarterial sympathetic nerves, Cardiology, Diseases, Medical research

## Abstract

Drug-coated balloon (DCB) therapy promotes beneficial vascular remodeling after percutaneous coronary intervention. However, its mechanisms remain elusive. Given paclitaxel’s neurotoxicity, the effects of paclitaxel (PTX)-DCB treatment on periarterial innervation are unknown. This study assessed the impact of PTX-DCB on periarterial innervation in porcine coronary arteries. Coronary arteries from twelve pigs were treated with balloon catheters across four groups: (1) uncoated balloon (POBA), (2) balloon with 3 µg PTX/mm^2^ (1 × 3), (3) balloon with 6 µg PTX/mm^2^ (1 × 6) and (4) two balloons each with 6 µg PTX/mm^2^ (2 × 6). Quantitative coronary angiography was performed before, during, and after balloon angioplasty, and at day 28 prior to sacrifice. Following sacrifice, tissue samples were taken for histological examination of periarterial nerve fibres. High-dose PTX treatment resulted in late lumen enlargement (+ 0.29 ± 0.60 mm) in the control angiography after 28d compared to POBA treatment (POBA vs. 2 × 6-PTX group (*p* = 0.0236). Sympathetic nerve fiber density did not differ between the untreated and balloon-treated segments in the POBA group (untreated: 386.5 ± 169.1 nerves/cm^2^, balloon: 328.5 ± 289.9 nerves/cm^2^, *p* = 0.6439) but was significantly reduced in the PTX-treated animals (1 × 3-PTX: untreated: 540.9 ± 216.2 nerves/cm^2^, balloon: 177.0 ± 130.5 nerves/cm^2^, *p* = 0.0020; 1 × 6-PTX: untreated: 546.5 ± 226.8 nerves/cm^2^, balloon: 142.4 ± 61.7 nerves/cm^2^, *p* = 0.0003; 2 × 6-PTX: untreated: 498.6 ± 404.0 nerves/cm^2^, balloon: 176.5 ± 103.4 nerves/cm^2^, *p* = 0.0478). PTX-DCB treatment significantly reduced the proportion of periarterial nerve fibers < 500 µm^2^ in the balloon-treated segment (*p* vs. untreated segment: POBA = 0.0267; 1 × 3-PTX = 0.0680; 1 × 6-PTX = 0.0028; 2 × 6-PTX = 0.0391). PTX-DCB therapy exerts previously unrecognized pleiotropic effects by reducing perivascular nerve fiber density, resulting in coronary denervation that may contribute to the luminal gain beyond antiproliferative effects.

**Supplementary Information:**

The online version contains supplementary material available at 10.1038/s41598-026-54572-3.

## Introduction

For most patients, percutaneous coronary intervention is the procedure of choice for myocardial revascularization^1^. Unlike earlier bare-metal stents, which often led to neointimal hyperplasia, the local delivery of antiproliferative substances such as paclitaxel (PTX) via drug-eluting stents (DES) or drug-coated balloons (DCB) has proven effective in preventing local intravascular restenosis after percutaneous coronary intervention, due to their liposomal nature and binding to various cellular components^2^. Nevertheless, lumen loss can occur over time following DES implantation, and the risk of device-related complications increases with the number and length of DES^3^. A salient feature of DCB therapy, particularly with PTX-coated devices, is its capacity to not only compensate for lumen loss but also promote late lumen enlargement (LLE) within the first year after treatment^4,5^. However, the mechanisms leading to this beneficial vascular remodeling of DCB treatment remain elusive.

Coronary arteries are densely innervated^6,7^ and the autonomic nervous system plays a key role in cardiac function and cardiovascular homeostasis^8^. Emerging evidence suggests that perivascular innervation around atherosclerotic lesions also contributes to maintenance and progression of atherosclerosis^9^. Given the neurotoxicity of PTX^10^, we hypothesized that PTX delivered via DCB may affect sympathetic nerve fibers surrounding coronary arteries. As coronary artery innervation regulates vasomotion^11^, reducing sympathetic vasoconstrictive effects through DCB treatment could partially explain the observed LLE and may help stabilize coronary artery disease by interrupting perivascular sympathetic signaling. Therefore, this study aimed to investigate the effects of balloon angioplasty and varying PTX concentrations delivered via DCB on periarterial sympathetic innervation in porcine coronary arteries.

## Methods

### Animal care

The animal experiments were approved according to all applicable institutional and national guidelines (EU Commission Directives 86/609/EEC, 2010/63EC and German Animal Protection Act) and were approved by the local animal ethics committee (Sachsen-Anhalt, Germany). All experiments were performed in accordance with relevant guidelines and regulations and are reported in accordance with the ARRIVE guidelines. Conventional farm pigs (castrated males, average weight 24–29 kg), purchased from a commercial pig farm, were studied for percutaneous transluminal coronary angioplasty and for drug release and transfer. All animals received equal treatment following standard protocols for anaesthesia and interventional procedures consistent with previous studies^12^. For angiography and euthanasia, animals were sedated with ketamine (Ursotamin; Serumwerk Bernburg, Germany; 0.2 mL/kg) and xylazine hydrochloride 2% (Xylazin; Riemser Arzneimittel GmbH, Germany; 0.1 mL/kg). General anaesthesia was induced by intravenous propofol (Recofol 1%; Curamed Pharma GmbH, Germany; 6–10 mL) followed by intramuscular meloxicam (Metacam®; Boehringer Ingelheim Vetmedica, Ingelheim am Rhein, Germany; 0.4 mL/kg) and intravenous butorphanol (Morphasol 10 mg/mL; aniMedica GmbH, Germany; 0.1 mg/10 kg) for analgesia. Animals were then endotracheally intubated (Endonorm; Rüsch GmbH, Germany) and mechanically ventilated using a gas mixture of 30–60 vol% oxygen and 40–70 vol% air with isoflurane (Isofluran Curamed; Curamed Pharma GmbH, Germany; 1–4%, adjusted to the depth of anaesthesia). Euthanasia was performed under deep anaesthesia (stage III, plane 4 according to Guedel) by intravenous bolus administration of 10 mL supersaturated potassium chloride. During the intervention, ECG, heart rate and oxygen saturation were continuously monitored. Blood pressure was measured before and after intervention.

### Experimental design and procedures: drug transfer

Coronary arteries from twelve pigs were treated with stent (3.0–8 mm, Rebel Monorail, Boston Scientific, USA) and balloon catheters (3.5–20 mm) in a randomised order. The balloons were placed fully covering the stent and in the vessel segment proximal of the stent. Four treatment groups were investigated: (1) uncoated balloon (POBA, control), (2) balloon with 3 µg paclitaxel/mm^2^ (IN.PACT™ Falcon), (3) balloon with 6 µg paclitaxel/mm^2^ (1 × 6, study device) and (4) two balloons each with 6 µg paclitaxel/mm^2^ (2 × 6, study device). The control examination was performed after four weeks.

### Quantitative coronary angiography

**Fig. 1:**
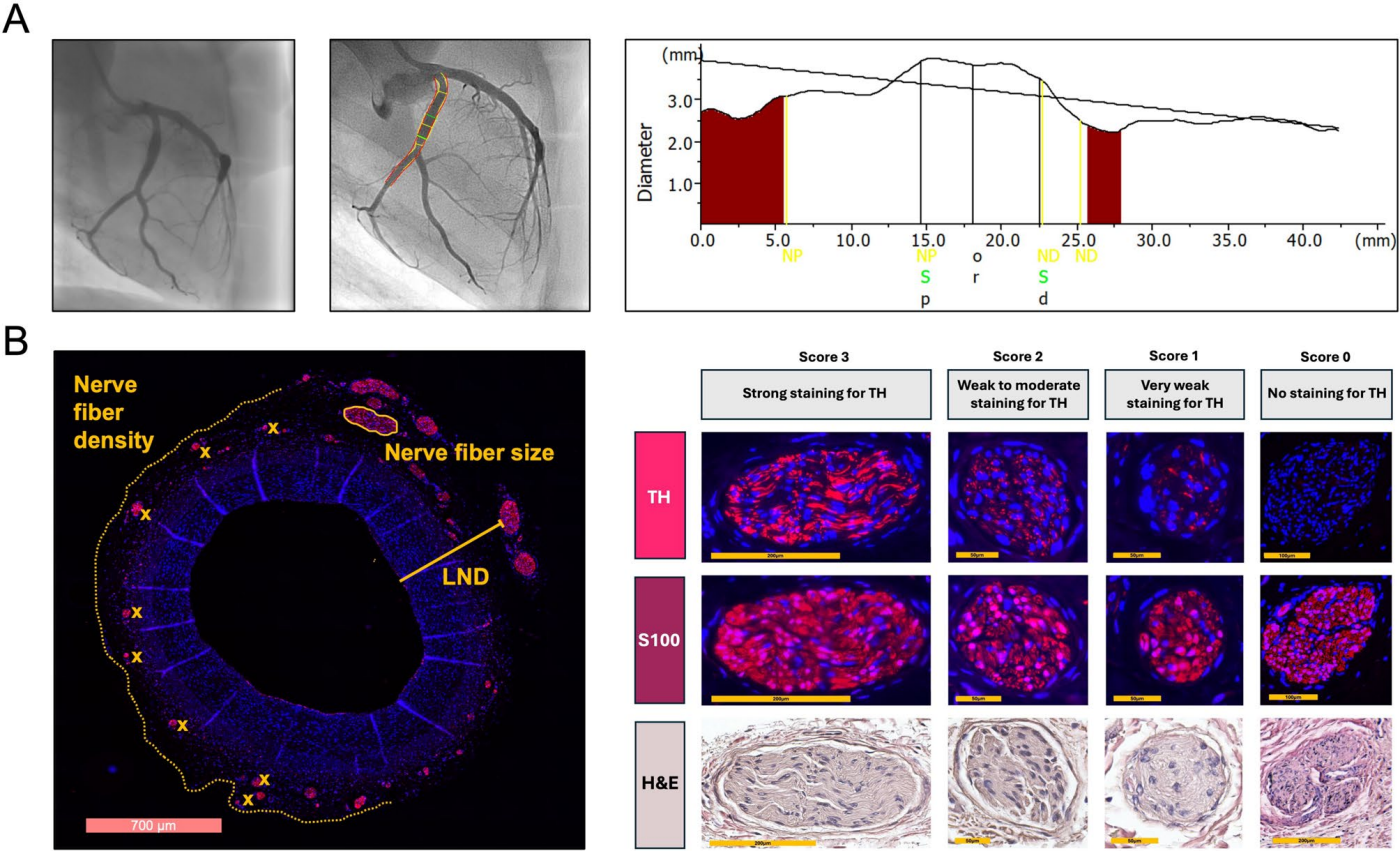
Representative images of quantitative coronary angiography, analysis methods of periarterial nerve fibers and semiquantitative scoring system for tyrosine hydroxylase immunostaining. (**A**) Angiography of the left coronary artery after 1 × 6 paclitaxel-DCB treatment. Reference lumen diameters (red line) were calculated using an automated contour detection tool, generating a hypothetical optimal vessel contour. The actual vessel diameter is indicated by the yellow line, while the balloon intervention was performed within the green-marked area. In the corresponding diagram (right), the reference vessel is displayed as a constant line, with a decrease in lumen diameter along the vessel course. The actual lumen diameter is shown as a curved line, demonstrating lumen enlargement in the treated segment. (**B**) Left panel: histological methods for quantifying periarterial nerve fibers. Right panel: representative perivascular nerve fibers with varying degrees of tyrosine hydroxylase immunostaining, together with the corresponding S100 and H&E staining. The semiquantitative scoring system was adapted from^15^.

Quantitative coronary angiography (QCA) was performed before, during, and after balloon angioplasty and before sacrifice (28-day follow-up). Analysis was performed with the QAngio XA System (Medis, Netherlands) blinded to treatment group (Fig. 1A). An angiographic image with minimal overlap from other vessels and optimal contrast enhancement was selected for QCA analysis. Minimum and mean lumen diameters were assessed before and after the procedure and at follow-up. Overstretch, late lumen loss and percent of stenosis were calculated.

### Tissue collection and staining

Four weeks after treatment, pigs were sacrificed and coronary segments harvested. Stent-treated vessels were embedded in methyl methacrylate and used for histomorphometry, inflammation, and injury assessment. Balloon-treated segments and directly proximal untreated controls were paraffin-embedded. For analysis, one section from the central region of each paraffin block was evaluated. All paraffin cross-sections underwent H&E staining (Morphisto) for morphology assessment. Neuronal tissue and nerve fiber distribution were assessed by S100 (Dako), sympathetic fibers by anti-tyrosine hydroxylase (TH) (Novusbio), and endo-/perineural fibrosis by Sirius Red (Morphisto). Representative sections are shown in Supplementary Fig. 1. To further assess medial architecture and elastic lamina integrity, additional Elastica van Gieson (EVG, Morphisto) staining was performed on balloon-treated sections from each treatment group.

### Histomorphometry

Quantitative histomorphometric measurements of the three sections were performed using NIS-Elements BR (V. 3.0, Nikon Instruments Inc., Tokyo, Japan). The following parameters were evaluated: lumen diameter, maximum neointimal thickness, lumen area, medial area, external elastic membrane area and neointimal area. The injury score was assigned as described by Schwartz^13^ and the inflammation score was graded as described by Kornowski^14^. In stent treated segments, inflammation score was assigned for each stent strut within the examined section. In balloon-treated segments, the score of the worst affected area was applied to avoid underestimating focal but relevant changes.

### Histological analysis of periarterial nerve fibers

**Fig. 2:**
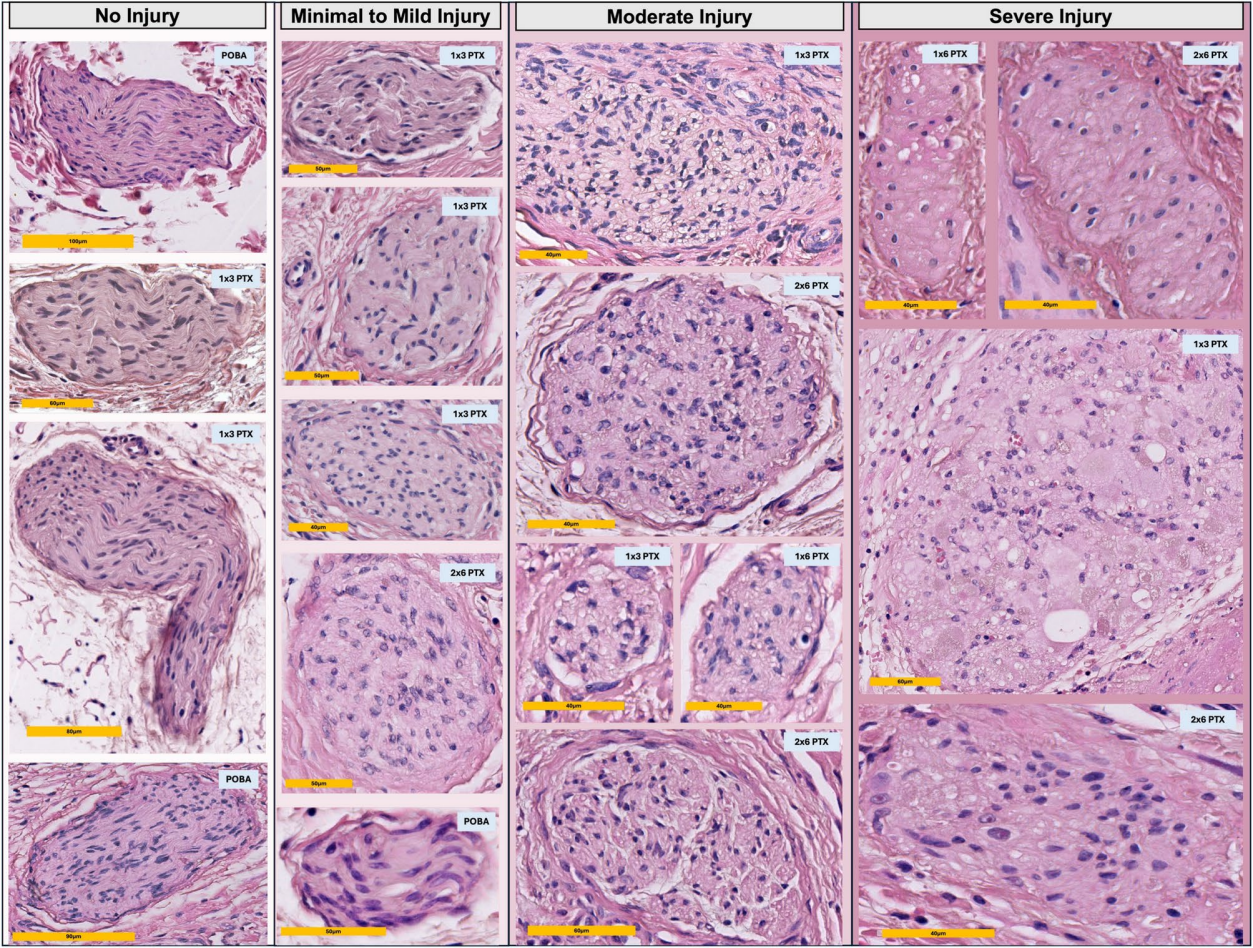
Semiquantitative grading scheme of periarterial nerves and treatment-related changes. Representative nerve fibers from all groups in the balloon-treated regions. Treatment-related changes were assessed using a semiquantitative scoring system^15^. Nerve fibers without injury showed no abnormalities. Minimal to mild injury was characterized by slight inflammation and fibrosis of the epi-, peri-, and endoneurium as well as mild vacuolization. In the moderate injury group, these alterations were more pronounced. Severe injury was associated with marked inflammation and fibrosis, necrosis, and complete effacement of nerve architecture.

Stained vessels were scanned using a whole slide scanner (Leica Aperio Versa 8, Leica Biosystems, Wetzlar, Germany) for digital slide generation. The digital pathology software Aperio ImageScope (V.12.4.6.5003, Leica Biosystems, Wetzlar, Germany) was used to analyze nerve fiber distribution (Fig. 1B). All sections were systematically evaluated using a semiquantitative scoring system^15^ adapted from renal denervation studies to assess TH immunostaining and perivascular sympathetic nerve injury. In the present coronary model, functional sympathetic denervation was conservatively defined as absent or minimal TH immunoreactivity, corresponding to scores 0–1 (Fig. 1B). Nerve fibers with a score of 2 were considered to show partially preserved TH expression and were therefore not classified as complete functional loss, in order to avoid overestimating denervation in a scoring system that has not yet been independently validated for coronary periarterial nerves. The number of nerve fibers, the size of nerve fibers and the shortest distance of nerve fibers to the luminal intima were analyzed for both stains. Nerve fiber density was calculated as the number of identified S100- or TH-positive periarterial nerve fibers divided by the total histological tissue area available for analysis on each cross-section and expressed as nerves/cm^2^. H&E-stained sections were used to investigate treatment effects on periarterial nerve fibers across the different groups. A semiquantitative grading scheme for periarterial nerves and treatment-related changes was used^15^ (Fig. 2).

### Statistical analysis

Normal distribution was assessed using the Shapiro–Wilk test. Results were expressed as mean ± SD. Unpaired t tests were used for comparisons between untreated and treated segments. Statistical comparison between treatment groups were evaluated by one-way ANOVA with Bonferroni’s post-hoc test for multiple comparison. A 2-tailed *p*-value < 0.05 was considered statistically significant. GraphPad Prism (Version 10.0.1, GraphPad Software, Boston, USA) was used for statistical analyses. Exploratory per-vessel Pearson correlations were used to assess associations between TH-positive nerve fiber loss and late lumen change, defined as minimal lumen diameter at 28-day follow-up minus post-interventional minimal lumen diameter. TH-positive nerve fiber loss was calculated as the difference in TH-positive nerve fiber density between untreated reference and balloon-treated segments. Baseline TH-positive nerve fiber density was additionally correlated with untreated lumen area.

## Results

### Quantitative coronary angiography (QCA)

Eight vessels per group (3 animals each) including LAD, LCX, RCA) were analyzed angiographically. Pre-interventional lumen diameters did not differ significantly between groups in either the stented or balloon-treated segments. In stented segments, mean pre-interventional luminal diameter was 2.19 ± 0.26 mm in the POBA group, 2.29 ± 0.25 mm in the 1 × 3 PTX group, 2.32 ± 0.35 mm in the 1 × 6 PTX group and 2.53 ± 0.50 mm in the 2 × 6 PTX group (overall ANOVA *p* = 0.2027), respectively. Post-intervention, lumen diameters increased in all groups without significant differences. The post-interventional lumen diameter was 3.10 ± 0.34 mm in the POBA group, 2.96 ± 0.32 mm in the 1 × 3 PTX group, 3.06 ± 0.29 mm in the 1 × 6 PTX group and 3.31 ± 0.42 mm in the 2 × 6 PTX group (overall ANOVA *p* = 0.2314), respectively. Based on the post-interventional minimal lumen diameter, late lumen loss was 1.00 ± 0.55 mm in the POBA group, 0.44 ± 0.54 mm in the 1 × 3 PTX group, − 0.11 ± 0.19 mm in the 1 × 6 PTX group, i.e. a lumen gain of 0.11 mm, and 0.06 ± 0.28 in the 2 × 6 PTX group, with a statistically significant difference between POBA vs. 1 × 6 PTX group (*p* < 0.0001) and POBA vs. 2 × 6 PTX group (*p* = 0.0007). A detailed summary of all QCA parameters measured in the stented segment, including statistical results, is summarized in Supplementary table 1.

In balloon treated segments, mean pre-interventional lumen diameter was 2.42 ± 0.21 mm in the POBA group, 2.50 ± 0.25 mm in the 1 × 3 PTX group, 2.57 ± 0.34 mm in the 1 × 6 PTX group and 2.75 ± 0.45 mm in the 2 × 6 PTX group (overall ANOVA *p* = 0.2339). Post-intervention, lumen diameters increased in all groups without significant differences. The post-interventional lumen diameter was 2.65 ± 0.15 mm in the POBA group, 2.85 ± 0.23 in the 1 × 3 PTX group, 2.82 ± 0.24 in the 1 × 6 PTX group and 2.90 ± 0.41 in the 2 × 6 PTX group (ANOVA overall *p* = 0.3025). Based on the post-interventional minimal lumen diameter, late lumen loss was 0.29 ± 0.37 mm in the POBA group, 0.48 ± 0.20 mm in the 1 × 3 PTX group, 0.12 ± 0.20 mm in the 1 × 6 PTX group and − 0.29 ± 0.60 mm in the 2 × 6 PTX group, i.e. a lumen gain of 0.29 mm, with a statistically significant difference between the 1 × 3 PTX vs. 2 × 6 PTX group (*p* = 0.0019) and POBA vs. 2 × 6 PTX group (*p* = 0.0236). A detailed summary of all QCA parameters measured in the balloon region, including statistical results, is summarized in Table 1.

**Table 1:** Lumen diameter, lumen area and neointimal thickness in the balloon treated segment.

Treatment group	POBA	1 × 3 PTX	1 × 6 PTX	2 × 6 PTX	*p*-overall	*p*-groups					
Analyzed vessel, n	8	8	8	8	*p* (Anova)	*p*: 1 × 3 PTX vs. POBA	*p*: 1 × 3 PTX vs. 1 × 6 PTX	*p*: 1 × 3 PTX vs. 2 × 6 PTX	*p*: POBA vs. 1 × 6 PTX	*p*: POBA vs. 2 × 6 PTX	*p*:1 × 6 PTX vs. 2 × 6 PTX
Mean lumen diameter pre intervention, mm	2.42 ± 0.21	2.50 ± 0.25	2.57 ± 0.34	2.75 ± 0.45	0.2339	0.9588	0.9743	0.4279	0.7946	0.1984	0.6812
Mean lumen diameter post intervention, mm	2.65 ± 0.15	2.85 ± 0.23	2.82 ± 0.24	2.90 ± 0.41	0.3025	0.4747	0.9974	0.9805	0.5904	0.2756	0.9389
Minimal lumen diameter post intervention, mm	2.48 ± 0.18	2.69 ± 0.26	2.68 ± 0.22	2.57 ± 0.57	0.5776	0.6198	> 0.9999	0.8945	0.6553	0.9547	0.9160
Mean lumen diameter at 28d follow up, mm	2.34 ± 0.31	2.56 ± 0.29	2.82 ± 0.25	3.22 ± 0.63	*0.0010	0.6995	0.5616	*0.0128	0.0998	*0.0008	0.2120
Minimal lumen diameter at 28d follow up, mm	2.18 ± 0.30	2.20 ± 0.26	2.55 ± 0.29	2.85 ± 0.59	*0.0044	0.9996	0.2854	*0.0113	0.2412	*0.0087	0.4241
Overstretch ratio	1.10 ± 0.12	1.14 ± 0.07	1.11 ± 0.13	1.06 ± 0.10	0.4926	0.8634	0.9269	0.4183	0.9984	0.8634	0.7816
Late lumen loss, mm	0.29 ± 0.37	0.48 ± 0.20	0.12 ± 0.20	− 0.29 ± 0.60	*0.0027	0.7481	0.2496	*0.0019	0.8055	*0.0236	0.1591
Percent of stenosis, %	17.28 ± 9.25	19.99 ± 9.63	4.57 ± 10.09	− 4.14 ± 22.00	*0.0050	0.9791	0.1395	*0.0083	0.2771	*0.0215	0.5947

Values are mean ± standard deviation or counts n. One-way ANOVA was used to test statistical significance. Bonferroni’s post-hoc test was used for multi comparisons. POBA—Plain old balloon angioplasty. 1 × 3 PTX—3 µg paclitaxel/mm^2^ balloon surface. 1 × 6 PTX—6 µg paclitaxel/mm^2^ balloon surface. 2 × 6 PTX—Two balloons with 6 µg paclitaxel/mm^2^ balloon surface**.** Significant results are asterisked.

### Histomorphometry

In the stented segment, lumen diameter and area increased with increasing amounts of PTX compared to the POBA group (POBA: 1.85 ± 0.57 mm, 3.09 ± 1.84 mm^2^; 1 × 3 PTX group: 2.32 ± 0.50 mm, 4.49 ± 1.68 mm^2^; 1 × PTX: 2.75 ± 0.23 mm, 5.87 ± 1.07 mm^2^; 2 × PTX: 2.81 ± 0.33 mm, 6.09 ± 1.73 mm^2^) (lumen diameter: *p* vs. POBA: 1 × 3 PTX = 0.1613; 1 × 6 PTX = 0.0015; 2 × 6 PTX = 0.0011) (Supplementary Fig. 2A & B). The effects on luminal narrowing were associated with stent induced neointimal growth. Neointimal thickness in POBA treated vessels was significantly greater (1.01 ± 0.36 mm) than in the PTX groups (1 × 3 PTX: 0.80 ± 0.42 mm; 1 × 6 PTX: 0.29 ± 0.06 mm; 2 × 6 PTX: 0.40 ± 0.17 mm) (*p* vs POBA: 1 × 3 PTX = 0.5056; 1 × 6 PTX = 0.0002; 2 × 6 PTX = 0.0026) (Supplementary Fig. 2C).

**Fig. 3:**
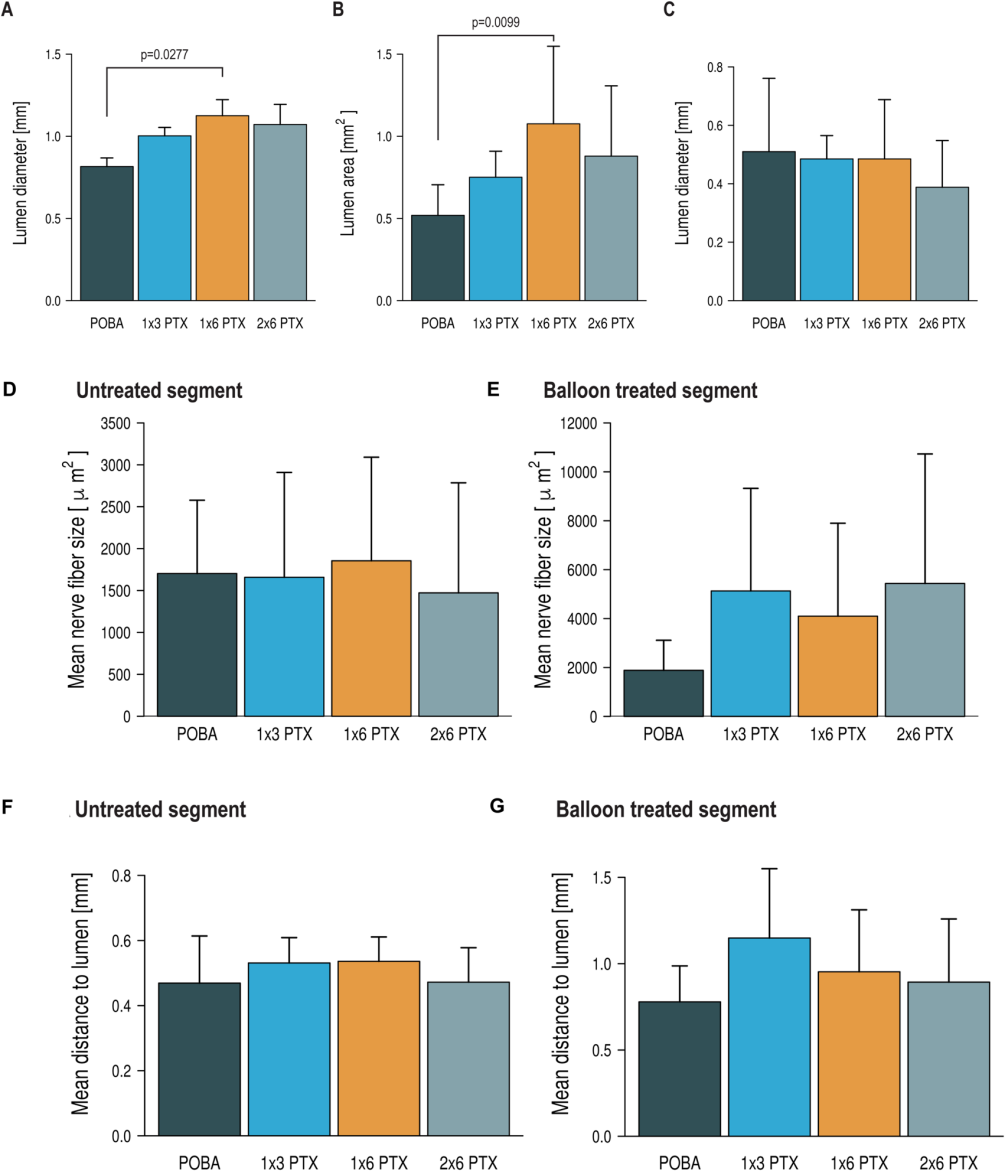
Histomorphometric parameters in the balloon-treated segment and quantitative comparison of nerve fiber size and distance in the untreated and balloon-treated segments. Lumen diameter (**A**), lumen area (**B**), and neointimal thickness (**C**) in the balloon-treated segment between the four treatment groups. Mean nerve fiber size in the untreated (**D**) and balloon-treated (**E**) segments and mean distance of periarterial nerve fibers to arterial lumen in the untreated (**F**) and balloon-treated (**G**) segments between the four treatment groups. Statistical significance of Bonferroni’s post-hoc test for multi comparison between individual groups is indicated by a bracket and the corresponding *p*-value. Error bars represent the standard deviation.

A similar pattern was observed in balloon-treated segments with increasing lumen diameter and area in the PTX groups (POBA: 0.83 ± 0.11mm, 0.53 ± 0.18 mm^2^; 1 × 3 PTX: 1.01 ± 0.11mm, 0.76 ± 0.15 mm^2^; 1 × 6 PTX: 1.13 ± 0.25 mm, 1.09 ± 0.46 mm^2^; 2 × 6 PTX: 1.08 ± 0.29 mm, 0.89 ± 0.42 mm^2^) (lumen diameter: *p* vs POBA: 1 × 3 PTX = 0.2930; 1 × 6 PTX = 0.0277; 2 × 6 PTX = 0.1025) (Fig. 3A,B). Similarly, a numerically decrease in neointima thickness was observed with increasing PTX content (POBA: 0.52 ± 0.24 mm, 1 × 3 PTX: 0.49 ± 0.08 mm; 1 × 6 PTX: 0.49 ± 0.20 mm; 2 × 6 PTX: 0.40 ± 0.15 mm) (*p* vs. POBA: 1 × 3 PTX = 0.9924; 1 × 6 PTX = 0.9934; 2 × 6 PTX = 0.5658) (Fig. 3C). Quantification of external elastic membrane area showed numerically larger total vessel areas in all PTX-DCB groups compared with POBA: 1.75 ± 0.45 mm^2^ in POBA, 2.56 ± 0.53 mm^2^ in 1 × 3 PTX, 2.87 ± 1.15 mm^2^ in 1 × 6 PTX, and 2.57 ± 0.91 mm^2^ in 2 × 6 PTX. The overall ANOVA showed a trend toward significance (*p* = 0.0577). Bonferroni-adjusted post-hoc comparisons showed a significantly larger external elastic membrane area in the 1 × 3 PTX group compared with POBA (*p* = 0.0306), whereas the comparisons of POBA vs. 1 × 6 PTX (*p* = 0.1347) and POBA vs. 2 × 6 PTX (*p* = 0.2306) did not reach statistical significance. Details of all histomorphometric parameters analyzed in the balloon treated segment are shown in Supplementary table 2. A summary of all histomorphometric parameters analyzed in the untreated and stent treated segment are shown in Supplementary tables 3 and 4. Inflammation and injury scores were comparable across groups (≈3) in the stent and balloon treated segments, indicating high-grade inflammation and vessel injury. Additional EVG staining demonstrated recognizable medial architecture across POBA, 1 × 3 PTX, 1 × 6 PTX, and 2 × 6 PTX-treated vessels, despite procedure-related vascular injury (Supplementary Fig. 3). The internal elastic lamina was mostly continuous, although focal irregularities or limited disruptions were occasionally observed. These focal changes were not restricted to PTX-treated arteries, were not preferentially enriched in higher-dose PTX groups, and did not show a dose-dependent pattern. Together with the comparable injury and inflammation scores across treatment groups, these findings do not support systematic medial destruction or IEL injury as the primary explanation for the observed favorable lumen remodeling. In balloon treated segments, degenerative changes of periarterial nerve fibers consistent with perineural and endoneural injury were observed, including vacuolization and the formation of digestion chambers. In severely affected fibers, necrosis and complete dissolution of neural structures were evident. Uninjured fibers were found predominantly in the POBA group. Nevertheless, even in these animals, minimal to mild injury was frequently present, which likely reflects the mechanical impact of high-pressure balloon inflation on the surrounding tissue. In contrast, nerve injury graded as moderate or severe was observed almost exclusively in PTX-treated areas (Fig. 2). Additionally, increased perineural and endoneurial fibrosis was observed in the PTX-treated areas (Supplementary Fig. 4).

### Periarterial nerve fiber size

The untreated vessel sections showed comparable mean nerve fiber sizes in the four treatment groups (POBA: 1720.5 ± 856.3 µm^2^; 1 × 3 PTX: 1679.7 ± 1228.5 µm^2^; 1 × 6 PTX: 1883.6 ± 1206.5 µm^2^; 2 × 6 PTX: 1486.2 ± 1300.6 µm^2^) (*p* vs. POBA: 1 × 3 PTX = 0.9999; 1 × 6 PTX = 0.9926; 2 × 6 PTX = 0.9811) (Fig. 3D).

**Fig. 4:**
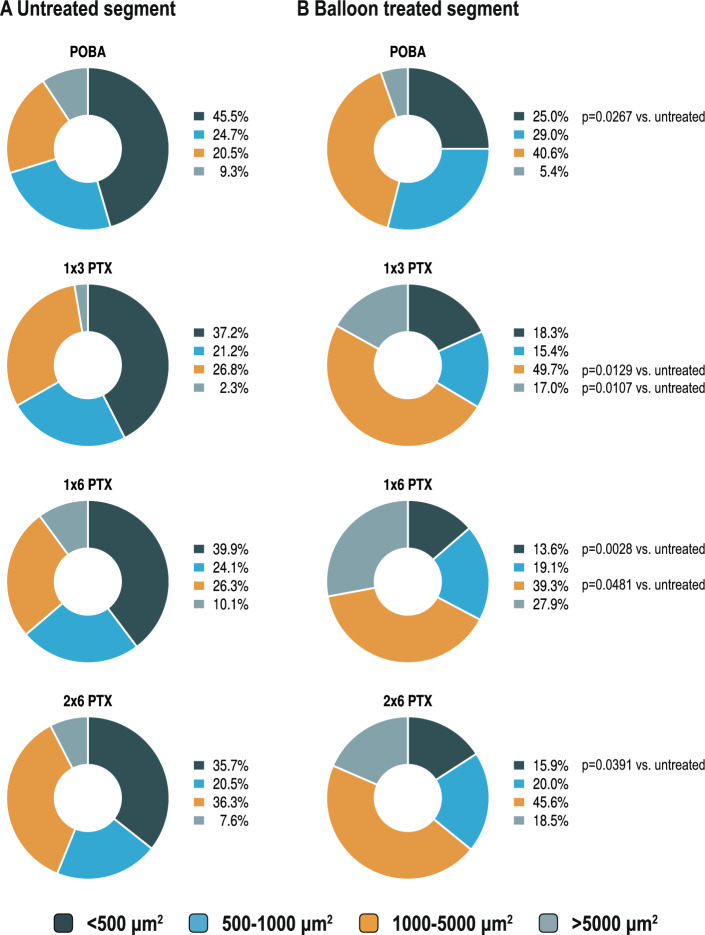
Percentage of nerve fiber size divided into categories. Percentage of nerve fiber size divided into four categories in the untreated (**A**) and balloon-treated (**B**) segments between the four treatment groups.

Compared to the untreated part of the vessel, nerve fiber size was larger in the PTX-treated groups. While the nerve fiber size in the POBA group was comparable to the untreated part of the vessel (1941.3 ± 1171.3 µm^2^), the area of the nerve fibers tended to increase with DCB treatment (1 × 3 PTX: 5223.4 ± 4103.8 µm^2^; 1 × 6 PTX: 4182.8 ± 3711.6 µm^2^; 2 × 6 PTX: 5517.3 ± 5215.7 µm^2^) (*p* vs. POBA: 1 × 3 PTX = 0.2336; 1 × 6 PTX = 0.5494; 2 × 6 PTX = 0.1785) (Fig. 3E). A direct comparison of nerve fiber size in the untreated and balloon treated segments within a treatment group showed a increase in mean nerve fiber size (*p* vs. untreated segment: POBA = 0.6730; 1 × 3 PTX = 0.0359; 1 × 6 PTX = 0.1451; 2 × 6 PTX = 0.1953). Dividing the periarterial nerve fibers into four categories according to their size (< 500 µm^2^, 500–1000 µm^2^, 1000–5000 µm^2^ and > 5000 µm^2^), it can be seen that mainly the proportion of small nerve fibers (< 500 µm^2^) is significantly reduced after balloon treatment (*p* vs. untreated segment: POBA = 0.0267; 1 × 3 PTX = 0.0680; 1 × 6 PTX = 0.0028; 2 × 6 PTX = 0.0391) (Fig. 4). Supplementary Fig. 5 presents representative nerve fibers from balloon-treated segments across the four groups. These fibers are visible in H&E staining but negative for TH staining, indicating functional nerve degeneration. This phenomenon was observed in all groups, with a more pronounced appearance in the PTX-treated regions. Details of the periarterial nerve fiber size in the untreated and balloon treated area are shown in Table 2.

**Table 2:** Comparison of quantitative parameters of coronary innervation between the untreated and treated segments in the four treatment groups and in S100- and TH-staining.

Treatment group	POBA		1 x 3 PTX		1 x 6 PTX		2 x 6 PTX	
S100-staining								
Vessel segment	Untreated	Balloon	Untreated	Balloon	Untreated	Balloon	Untreated	Balloon
Analyzed vessels, n	8	8	8	7	8	7	8	7
Nerve fiber density, nerves/cm^2^	581.7 ± 101.3	647.8 ± 497.3	594.0 ± 311.9	455.6 ± 257.3	650.2 ± 103.9	*318.7 ± 164.6	568.7 ± 297.8	350.1 ± 169.1
Distance to lumen, mm	0.44 ± 0.13	*0.79 ± 0.20	0.54 ± 0.07	*1.16 ± 0.39	0.54 ± 0.07	*0.97 ± 0.35	0.49 ± 0.09	*0.91 ± 0.35
Nerve fiber size, µm^2^	1720.5 ± 856.3	1941.3 ± 1171.3	1679.7 ± 1228.5	*5223.4 ± 4103.8	1883.6 ± 1206.5	4182.8 ± 3711.6	1486.2 ± 1300.6	5517.3 ± 5215.7
< 500 µm^2^, %	45.5 ± 15.5	*25.0 ± 17.5	37.2 ± 21.8	18.3 ± 16.0	39.9 ± 17.0	*13.6 ± 10.2	35.7 ± 19.6	*15.9 ± 13.6
500–1000 µm^2^, %	24.7 ± 6.5	29.0 ± 8.8	21.2 ± 9.7	15.4 ± 12.7	24.1 ± 5.4	19.1 ± 13.6	20.5 ± 10.0	20.0 ± 12.0
1000–5000 µm^2^, %	20.5 ± 12.7	40.6 ± 20.1	26.8 ± 16.5	*49.7 ± 15.7	26.3 ± 7.4	*39.3 ± 14.2	36.3 ± 12.1	45.6 ± 8.3
> 5000 µm^2^, %	9.3 ± 8.8	5.4 ± 4.3	2.3 ± 4.8	*17.0 ± 13.3	10.1 ± 9.0	27.9 ± 32.4	7.6 ± 13.3	18.5 ± 12.1
TH-staining								
Analyzed vessels, n	8	6	6	8	7	8	7	8
Nerve fiber density, nerves/cm^2^	386.5 ± 169.1	328.5 ±289.9	540.9 ± 216.2	*177.0 ± 130.5	546.5 ± 226.8	*142.4 ± 61.7	498.6 ± 404.0	*176.5 ± 103.4
Distance to lumen, mm	0.47 ± 0.14	*0.78 ± 0.19	0.53 ± .0.08	*0.98 ± 0.55	0.58 ± 0.10	*0.84 ± 0.29	0.48 ± 0.10	*0.63 ± 0.14
Nerve fiber size, µm^2^	2577.1 ± 1570.9	3959.3 ± 3538.5	1498.4 ± 1577.2	5596.7 ± 5049.5	2226.7 ± 1474.8	*4953.2 ± 2704.8	2795.9 ± 2659.4	*7245.1 ± 4538.6
< 500 µm^2^, %	23.2 ± 18.8	11.5 ± 17.8	31.0 ± 22.2	*9.8 ± 9.6	26.5 ± 18.4	*4.4 ± 9.0	23.3 ± 16.2	6.9 ± 14.4
500–1000 µm^2^, %	27.9 ± 23.6	19.9 ± 18.6	33.0 ± 32.7	22.2 ± 15.9	26.1 ± 9.8	*8.6 ± 6.0	30.5 ± 20.3	14.4 ± 12.9
1000–5000 µm^2^, %	34.2 ± 13.7	51.8 ± 25.2	23.3 ± 22.0	43.3 ± 18.9	39.4 ± 12.7	58.5 ± 20.3	38.9 ± 19.3	53.1 ± 13.8
> 5000 µm^2^, %	14.8 ± 13.2	16.8 ± 18.9	3.2 ± 6.9	24.7 ± 27.5	12.1 ± 12.5	28.5 ± 18.6	15.4 ± 22.9	25.7 ± 13.6

Values are mean ± standard deviation or counts n. Unpaired t tests were used for comparisons between untreated and treated segments. 1 × 3 PTX—3 µg paclitaxel/mm^2^ balloon surface. 1 × 6 PTX—6 µg paclitaxel/mm^2^ balloon surface. 2 × 6 PTX—Two balloons with 6 µg paclitaxel/mm^2^ balloon surface. Significant results are asterisked. Exact *p*-values are given in Supplementary table 5.

### Periarterial nerve fiber distance to arterial lumen

The periarterial nerve fibers of the untreated vessel segments showed comparable mean distance to the arterial lumen in the four treatment groups (POBA: 0.44 ± 0.13 mm; 1 × 3 PTX: 0.54 ± 0.07 mm; 1 × 6 PTX: 0.54 ± 0.07 mm; 2 × 6 PTX: 0.49 ± 0.09 mm) (*p* vs. POBA: 1 × 3 PTX = 0.7017; 1 × 6 PTX = 0.1945; 2 × 6 PTX = 0.9998) (Fig. 3F).

Compared to the untreated part of the vessel, the distance of the periarterial nerve fibers to the arterial lumen significantly increased in the balloon treated segments in each group (POBA: 0.79 ± 0.20 mm; 1 × 3 PTX: 1.16 ± 0.39 mm; 1 × 6 PTX: 0.97 ± 0.35 mm; 2 × 6 PTX: 0.91 ± 0.35 mm) (*p* vs. untreated segment: POBA = 0.0009; 1 × 3 PTX = 0.0005; 1 × 6 PTX = 0.0073; 2 × 6 PTX = 0.0092) (Fig. 3G). Details of the periarterial nerve fiber distance to arterial lumen in the untreated and balloon treated area are shown in Table 2.

### Periarterial nerve fiber density

In the untreated area, no significant differences in S100-positive nerve fibers density were observed between the four treatment groups (POBA: 581.7 ± 101.3 nerve/cm^2^; 1 × 3 PTX: 594.0 ± 311.9 nerve/cm^2^; 1 × 6 PTX: 650.2 ± 103.9 nerve/cm^2^; 2 × 6 PTX: 568.7 ± 297.8 nerve/cm^2^) (*p* vs. POBA: 1 × 3 PTX = 0.9995; 1 × 6 PTX = 0.9371; 2 × 6 PTX = 0.9995). In the balloon treated area, PTX treatment numerically reduced nerve fiber density (POBA: 647.8 ± 497.3 nerves/cm^2^; 1 × 3 PTX: 455.6 ± 257.3 nerves/cm^2^; 1 × 6 PTX: 318.7 ± 164.6 nerves/cm^2^; 2 × 6 PTX: 350.1 ± 169.1 nerves/cm^2^) (*p* vs. POBA: 1 × 3 PTX = 0.6424; 1 × 6 PTX = 0.2054; 2 × 6 PTX = 0.2822) (Supplementary Fig. 6A & C). Similar to the total nerve fiber density, no differences between groups were observed in TH-positive sympathetic nerve fibers in the untreated vessel segment (POBA: 386.5 ± 169.1 nerves/cm^2^; 1 × 3 PTX: 540.9 ± 216.2 nerves/cm^2^; 1 × 6 PTX: 546.5 ± 226.8 nerves/cm^2^; 2 × 6 PTX: 498.6 ± 404.0 nerves/cm^2^) (*p* vs. POBA: 1 × 3 PTX = 0.7122; 1 × 6 PTX = 0.6606; 2 × 6 PTX = 0.8498). In the balloon treated area, PTX treatment numerically reduced sympathetic nerve fiber density (POBA: 328.5 ± 289.9 nerves/cm^2^; 1 × 3 PTX: 177.0 ± 130.5 nerves/cm^2^; 1 × 6 PTX: 142.4 ± 61.7 nerves/cm^2^; 2 × 6 PTX: 176.5 ± 103.4 nerves/cm^2^) (*p* vs. POBA: 1 × 3 PTX = 0.3027; 1 × 6 PTX = 0.1515; 2 × 6 PTX = 0.2999) (Supplementary Fig. 6B & D).

### Comparison of balloon treated and untreated area

**Fig. 5:**
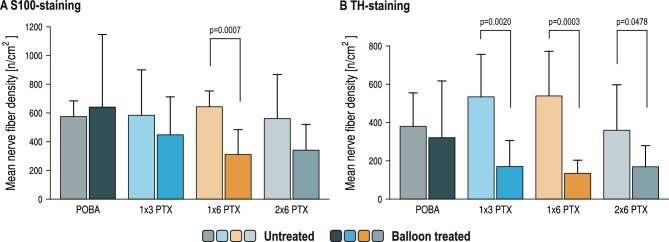
Comparison of mean nerve fiber density between the untreated and treated segments in the four treatment groups in S100-staining (**A**) and TH-staining (**B**). Statistical significance of Bonferroni’s post-hoc test for multi comparison between individual groups is indicated by a bracket and the corresponding *p*-value. Error bars represent the standard deviation.

Quantitative analysis of periarterial nerve fibers between treated and untreated segments in the different groups showed that nerve fiber density was reduced in the balloon treated segment. While the S100 nerve fiber density did not differ in the POBA group (untreated 581.7 ± 101.3 nerves/cm^2^, balloon 647.8 ± 497.3 nerves/cm^2^, *p* = 0.7186), the nerve fiber density decreased in the PTX treated segments (1 × 3 PTX: untreated: 594.0 ± 311.9 nerves/cm^2^, balloon: 455.6 ± 257.3 nerves/cm^2^, *p* = 0.3779; 1 × 6 PTX: untreated: 650.2 ± 103.9 nerves/cm^2^, balloon: 318.7 ± 164.6 nerves/cm^2^, *p* = 0.0007; 2 × 6 PTX: untreated: 568.7 ± 297.8 nerves/cm^2^, balloon: 350.1 ± 169.1 nerves/cm^2^, *p* = 0.1170) (Fig. 5A).

Similarly, TH-positive sympathetic nerve fiber density did not differ in the POBA group (untreated: 386.5 ± 169.1 nerves/cm^2^, balloon: 328.5 ± 289.9 nerves/cm^2^, *p* = 0.6439), but was significantly reduced in the PTX-treated animals (1 × 3 PTX: untreated: 540.9 ± 216.2 nerves/cm^2^, balloon: 177.0 ± 130.5 nerves/cm^2^, *p* = 0.0020; 1 × 6 PTX: untreated: 546.5 ± 226.8 nerves/cm^2^, balloon: 142.4 ± 61.7 nerves/cm^2^, *p* = 0.0003; 2 × 6 PTX: untreated: 498.6 ± 404.0 nerves/cm^2^, balloon: 176.5 ± 103.4 nerves/cm^2^, *p* = 0.0478) (Fig. 5B). Additionally, TH/S100 positive nerve fiber ratio significantly decreased after balloon treatment (POBA: untreated: 0.67 ± 0.26, balloon: 0.37 ± 0.18, *p* = 0.0188; 1 × 3 PTX: untreated: 1.26 ± 0.96, balloon: 0.40 ± 0.14, *p* = 0.0251; 1 × 6 PTX: untreated: 0.90 ± 0.52, balloon: 0.46 ± 0.21, *p* = 0.0441; 2 × 6 PTX: untreated: 0.90 ± 0.61, balloon: 0.46 ± 0.26, *p* = 0.0917) (Supplementary Fig. 7). Exploratory per-vessel analysis showed that greater reduction in TH-positive nerve fiber density tended to be associated with more favorable late lumen change, defined as less late lumen loss or greater lumen enlargement, but this relationship did not reach statistical significance (Pearson r = 0.195, *p* = 0.319) (Supplementary Fig. 8). In untreated reference segments, TH-positive nerve fiber density showed a numerical inverse trend with lumen area (Pearson r = -0.244, *p* = 0.211).

## Discussion

**Fig. 6:**
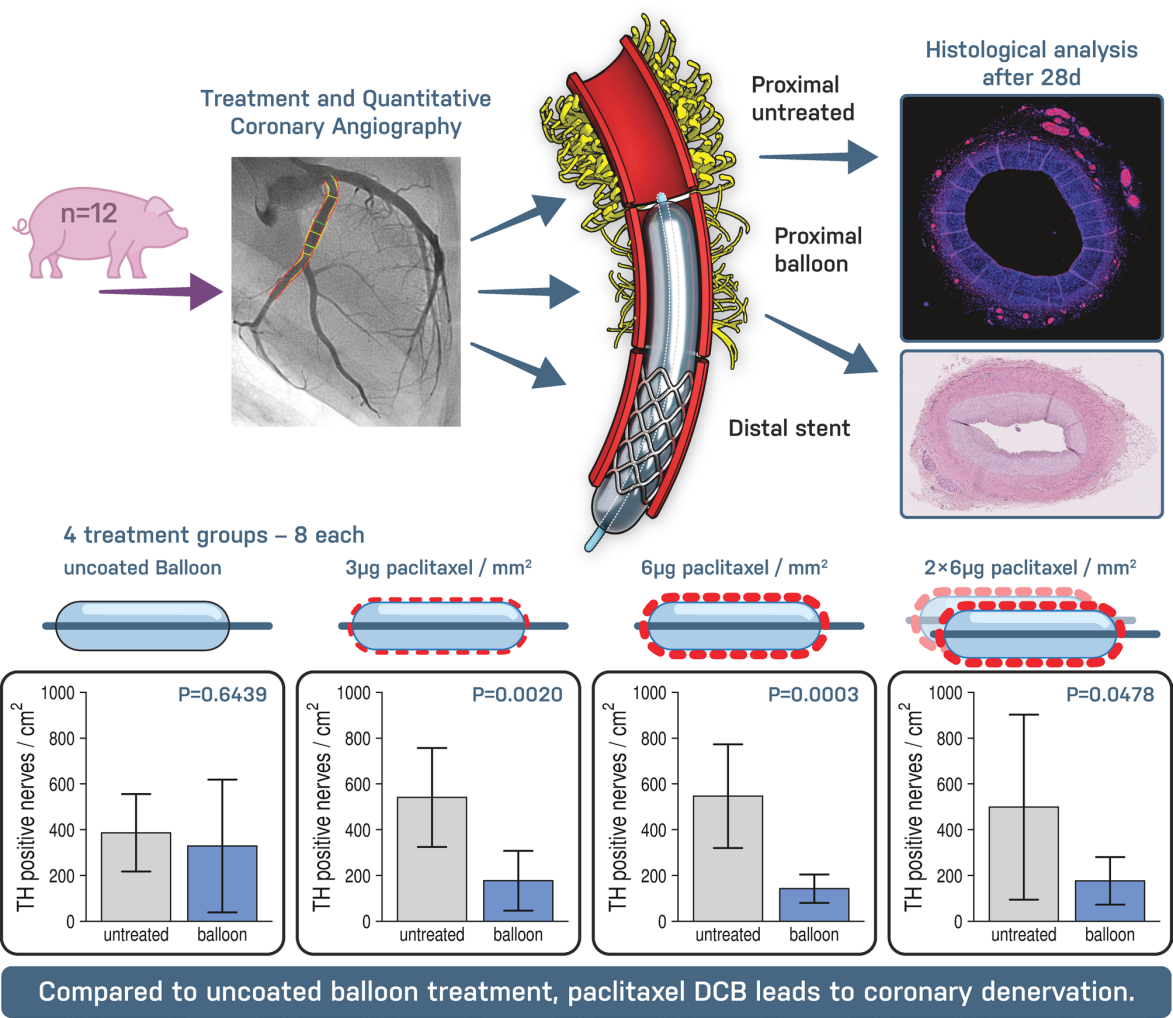
Schematic overview of the experimental design and main findings. Paclitaxel-coated balloon treatment reduced TH-positive periarterial sympathetic nerve fiber density in balloon-treated coronary segments after 28 days.

This study aimed to evaluate the effects of PTX-DCB on coronary innervation in a porcine model. We found that treatment of coronary arteries with PTX, both in stented and balloon treated segments resulted in an increase in lumen diameter compared to POBA treatment. PTX treatment was associated with less late lumen loss and lumen enlargement at follow-up compared to POBA treatment with increasing paclitaxel doses. Quantitative analysis of sympathetic coronary innervation showed a significant reduction in periarterial nerve fiber density in PTX-treated segments compared to untreated segments (Fig. 6). The present findings suggest that PTX-DCB treatment may result in sympathetic coronary denervation after four weeks in a porcine model.

Late lumen enlargement (LLE) of coronary arteries following DCB therapy is a well-recognized phenomenon^4,5^. LLE is characterized as an increase in vessel lumen diameter at follow-up exceeding the initial post-interventional lumen gain^4^. We observed a greater primary lumen gain in the stented segment than in the DCB treated segment due to mechanical expansion. However, DCB-treated segments may compensate through LLE within the first year after treatment, as previously described^16,17^. In the past, vessel wall dissection induced by balloon inflation was considered a negative side effect of DCB treatment. In contrast, Yamamoto et al. and others suggested that coronary dissection has no negative impact, but rather a favorable long-term outcome due to its positive influence on chronic vessel enlargement^18,19^. A potential benefit of aggressive primary treatment, including a higher dissection grade after PTX-DCB, has been postulated, which may result in improved initial lumen gain and reduced late lumen loss^20^. The mechanisms behind this positive vascular remodeling remain incompletely understood^21,22^. Quantification of the external elastic membrane area showed numerically larger total vessel areas in all PTX-DCB-treated groups compared with POBA, suggesting that luminal enlargement may partly reflect positive outward remodeling rather than suppression of neointimal hyperplasia alone. Despite procedure-related vascular injury, EVG analysis showed recognizable medial architecture and a mostly continuous internal elastic lamina across treatment groups. Occasional focal internal elastic lamina irregularities showed no PTX-specific or dose-dependent distribution. Together with comparable injury and inflammation scores, these findings argue against systematic medial destruction or internal elastic lamina injury as the primary mechanism of PTX-associated favorable lumen remodeling. Rather, they support the interpretation that periarterial sympathetic denervation may contribute to this effect.

Previous studies have shown that coronary arteries are circumferentially innervated by nerve fibers from the cardiac plexus^6,7^. PTX, used in DCB treatment for its antiproliferative effects on smooth muscle cells proliferation by arresting cells in the M phase of mitosis and blocking microtubule degradation^2^, accumulates in the adventitia^23^ and is known to exert neurotoxic effects^10^. Herein, nerve fiber density was shown to decrease with increasing PTX concentration up to 6 µg PTX/mm^2^ in the DCB-treated areas, especially comparing the treated with the untreated segments. However, this pattern was not observed in the POBA group. Morphological analysis revealed endoneural and perineural injury in both the POBA and PTX groups, while severe damage, including structural dissolution, necrosis and fibrosis, occurred predominantly in the PTX groups. These alterations resemble patterns reported in preclinical models of renal denervation^24^. Although coronary periarterial nerves are located closer to the arterial lumen than renal sympathetic nerves, PTX-DCB treatment should not be interpreted as a dedicated or necessarily more efficient neuromodulatory therapy. Rather, the observed reduction in sympathetic nerve fibers may represent a localized pleiotropic effect of PTX-DCB therapy. Secondary coronary denervation may therefore occur during routine clinical use, without an apparent denervation-related safety signal to date. In this context, given that coronary vasomotion is mediated by the sympathetic nervous system^11^, modulation of the periarterial innervation by DCB therapy may alter the response of the coronary arteries to sympathetic activation. Following sympathetic activation, stimulation of alpha_1_ receptors on coronary smooth muscle cells can lead to coronary vasoconstriction, while beta_1_ receptor stimulation promotes coronary vasodilation^25^. In atherosclerotic coronary arteries, increased sympathetic activation is associated with altered receptor regulation and consequent vasoconstriction^25^. By modulating perivascular innervation, DCB treatment in stenotic regions of patients with coronary artery disease may attenuate vasoconstriction, thereby contributing to late lumen enlargement and secondary luminal gain at follow-up. Furthermore, reducing perivascular sympathetic innervation might decrease the proliferation of smooth muscle cells caused by alpha-adrenergic signaling, which may also be a contributing factor^26^. Kawai et al. demonstrated that eight months after treatment of native lesions, the response to acetylcholine-induced vasoconstriction was less pronounced in the distal segments of DCB treated vessels than in those treated with a DES. While this has been attributed to better-preserved endothelial function, coronary denervation caused by DCB treatment could also account for the reduced vasoconstriction^27^. The observed increase in mean nerve fiber size in the PTX-treated segments likely reflects loss of small nerve fibers (< 500 µm^2^), while larger nerve fibers remain relatively unaffected. This finding may be physiologically relevant, as smaller periarterial fibers may represent distal autonomic effector branches that are positioned close to the vessel wall and contribute to local neurovascular regulation of coronary vasomotion. Thus, preferential loss of these small fibers could attenuate sympathetic vasoconstrictive signaling more directly than injury to larger proximal nerve bundles^6,11,25^. Proportionally, more sympathetic nerve fibers appeared to be affected by PTX-DCB treatment than other nerve fiber qualities, as indicated by the reduced TH/S100 ratio. Additionally, the increased distance between periarterial nerves and the vessel lumen likely results from neointimal thickening in the balloon-treated segments. In the exploratory per-vessel analysis, greater reduction in TH-positive nerve fiber density showed a non-significant trend toward more favorable late lumen change, reflecting less late lumen loss or greater lumen enlargement. Although directionally consistent with our hypothesis, this finding remains limited by the small sample size. Thus, DCB-associated LLE is likely multifactorial. Reduced sympathetic nerve fiber density may represent one contributing mechanism by modulating vasoconstrictive and vasomotor tone, alongside antiproliferative effects, vessel injury, inflammation, and structural remodeling. In untreated reference segments, TH-positive nerve fiber density showed a numerical inverse trend with lumen area, consistent with a potential influence of sympathetic innervation on coronary vasomotor tone. However, this finding remains exploratory and does not establish a functional or causal relationship.

The sympathetic nervous system contributes to the development and progression of atherosclerosis^28^. Recent studies identified neuroimmune-cardiovascular interfaces in the adventitia of atherosclerotic coronary segments^9^, where sympathetic fibers interact with immune cells and influence plaque formation^9^. In an atherosclerotic mouse model, celiac ganglionectomy disrupted cardiac sympathetic fibers, prevented disease progression, and stabilized plaques^9^. One may hypothesize that DCB-induced coronary denervation may provide benefits beyond late lumen enlargement, including potential stabilization of coronary artery disease. It might also represent a less invasive and safer alternative to thoracoscopic sympathectomy^29,30^ for sympathetically mediated conditions, such as refractory coronary spasm and severe ventricular arrhythmias, suggesting possible relevance for clinical translation of these findings.

### Study limitations

Although this large animal model closely resembles human coronary anatomy and physiology, it does not fully replicate human disease, as healthy non-atherosclerotic arteries were studied. The 28-day endpoint is standard in juvenile porcine coronary models and reflects accelerated vascular healing and neointimal formation. However, it does not directly correspond to the clinical time course of LLE. Therefore, the observed luminal gain should be interpreted as early positive remodeling in a preclinical model rather than as a direct equivalent of long-term clinical LLE. Longer-term effects on coronary innervation require further investigation. Because tissue paclitaxel concentrations were not measured, no quantitative dose–response relationship between drug concentration and neurotoxicity could be determined. Advanced atherosclerotic plaques may limit or alter paclitaxel diffusion toward periarterial nerves, potentially attenuating DCB-induced denervation in humans. However, because atherosclerotic plaques and the adjacent adventitia are sympathetically innervated neuroimmune vascular interfaces, modulation of periarterial sympathetic signaling may still be relevant in diseased coronary segments. Coronary periarterial innervation may vary according to coronary artery territory, thereby contributing to variability in baseline nerve-related parameters, including TH/S100-positive nerve fiber ratios, in untreated reference segments. Although the present study was not powered for formal vessel-specific comparisons, treatments were randomized across LAD, RCA, and LCX, and baseline angiographic and histomorphometric parameters were well matched between groups, minimizing the risk of systematic bias. Finally, exploratory per-vessel correlation analyses were limited by the small sample size and limited power. Since TH-positive nerve fiber density is only a structural marker, larger mechanistic studies with functional vasomotor testing are required.

## Conclusion

This study is the first to demonstrate that DCB therapy exerts previously unrecognized pleiotropic effects beyond the initial luminal gain by modulating perivascular nerve fibers, resulting in coronary denervation after four weeks in a porcine model. Further studies are needed to investigate the therapeutic potential and clinical implications of this novel mechanism.

## Supplementary Information

Supplementary Information.

## Data Availability

All data related to this study are available from the corresponding author on reasonable request.

## Abbreviations

DCB: Drug-coated balloon
DES: Drug-eluting stent
EVG: Elastica van Gieson
H&E: Hematoxylin and Eosin
LLE: Late lumen enlargement
PTX: Paclitaxel
TH: Tyrosine hydroxylase
