# Supplementary material for: Paclitaxel-coated balloon treatment is associated with sympathetic coronary denervation in a porcine model
Source: Sci Rep. 2026 Jun 4;16:17518. doi: 10.1038/s41598-026-54572-3 (PMC13241529; doi:10.1038/s41598-026-54572-3)
Supplement: Supplementary file 1 — Supplementary Information. [file 41598_2026_54572_MOESM1_ESM.docx]

**Supplementary Material**

**Paclitaxel-Coated Balloon Treatment Is Associated With Sympathetic Coronary Denervation in a Porcine Model**

Short Title: Impact of paclitaxel-DCB on coronary innervation

Mert Tokcan*****^1^, MD, Stephanie Bettink*****^2^, PhD, Ole Gemeinhardt^3^, DVM, Saarraaken Kulenthiran^1^, MD, Michael Böhm, MD, Felix Mahfoud^4,5^, MD, MA, Bruno Scheller^1,2^, MD

1. Klinik für Innere Medizin III – Kardiologie, Angiologie und Internistische Intensivmedizin, Saarland University Medical Center and Saarland University, Homburg, Germany
2. Clinical and Experimental Interventional Cardiology, Saarland University, Faculty of Medicine, Homburg, Germany
3. Department of Radiology, Charité - Universitätsmedizin Berlin, Corporate Member of Freie Universität Berlin and Humboldt-Universität zu Berlin, Berlin, Germany
4. Department of Cardiology, University Heart Center, University Hospital Basel, Switzerland
5. Cardiovascular Research Institute Basel (CRIB), University Heart Center, University Hospital Basel, Switzerland

*****These authors contributed equally to this work.

**Supplementary table 1:**

Summary of analyzed QCA parameters in the stent treated segment 4

**Supplementary table 2:**

Analysis of histomorphometric parameters in the balloon treated segment5

**Supplementary table 3:**

Analysis of histomorphometric parameters in the untreated segment6

**Supplementary table 4:**

Analysis of histomorphometric parameters in the stent treated segment7

**Supplementary table 5:**

P-values for comparison of quantitative parameters of coronary innervation 8

**Supplementary figure 1:**

Representative images of histological sections used for analyses 9

**Supplementary figure 2:**

Lumen diameter, lumen area and neointimal thickness in the stent treated segment 10

**Supplementary figure 3:**

EVG staining of balloon-treated coronary segments11

**Supplementary figure 4:**

Assessment of peri- and endoneural fibrosis across treatment groups 12

**Supplementary figure 5:**

Representative images of nerve fibers affected by the procedure 13

**Supplementary figure 6:**

Mean nerve fiber density in the untreated and balloon treated segments in S100- and TH-staining 14

**Supplementary figure 7:**

TH/S100 positive nerve fiber ratio15

**Supplementary figure 8:**

Per-vessel association between TH-positive nerve fiber loss and late lumen change 16

**Supplementary table 1: Summary of analyzed QCA parameters in the stent treated segment**

| Group | POBA | 1x3 PTX | 1x6 PTX | 2x6 PTX | p-overall | p-groups | | | | | |
| --- | --- | --- | --- | --- | --- | --- | --- | --- | --- | --- | --- |
| Analyzed vessel, n | 8 | 8 | 8 | 8 |  | 1x3 PTX vs. POBA | 1x3 PTX vs. 1x6 PTX | 1x3 PTX vs. 2x6 PTX | POBA vs. 1x6 PTX | POBA vs. 2x6 PTX | 1x6 PTX vs. 2x6 PTX |
| Mean lumen diameter pre Intervention, mm | 2.19±0.26 | 2.29±0.25 | 2.32±0.35 | 2.53±0.50 | 0.2027 | 0.7395 | >0.9999 | 0.6370 | 0.7705 | 0.1449 | 0.6032 |
| Mean lumen diameter post Intervention, mm | 3.10±0.34 | 2.96±0.32 | 3.06±0.29 | 3.31±0.42 | 0.2314 | 0.8216 | 0.9364 | 0.1877 | 0.9918 | 0.6304 | 0.4586 |
| Minimal lumen diameter post Intervention, mm | 2.79±0.31 | 2.77±0.39 | 2.81±0.29 | 3.04±0.37 | 0.3829 | 0.9996 | 0.9935 | 0.4172 | 0.9985 | 0.4757 | 0.5723 |
| Mean lumen diameter at 28d follow up, mm | 1.93±0.48 | 2.58±0.67 | 3.10±0.30 | 3.22±0.50 | *<0.0001 | 0.0726 | 0.1885 | 0.0749 | *0.0004 | *0.0001 | 0.9640 |
| Minimal lumen diameter at 28d follow up, mm | 1.79±0.56 | 2.33±0.62 | 2.92±0.26 | 2.98±0.45 | *<0.0001 | 0.1501 | 0.1002 | 0.0617 | *0.0005 | *0.0002 | 0.9954 |
| Overstretch ratio | 1.42±0.08 | 1.29±0.09 | 1.34±0.18 | 1.33±0.15 | 0.2569 | 0.2137 | 0.8975 | 0.9417 | 0.5711 | 0.4914 | 0.9992 |
| Late lumen loss, mm | 1.00±0.55 | 0.44±0.54 | -0.11±0.19 | 0.06±0.28 | *<0.0001 | 0.0597 | 0.0669 | 0.2893 | *<0.0001 | *0.0007 | 0.8611 |
| Stenosis diameter | 32.85±19.50 | 15.56±22.78 | -9.06±9.33 | -8,56±15.49 | *<0.0001 | 0.2214 | *0.0416 | *0.0474 | *0.0003 | *0.0003 | >0.9999 |

Values are mean±standard deviation. One-way ANOVA was used to test statistical significance. Bonferroni’s post-hoc test was used for multi comparisons. POBA – Plain old balloon angioplasty. 1x3 PTX – 3 µg paclitaxel/mm² balloon surface. 1x6 PTX – 6 µg paclitaxel/mm² balloon surface. 2x6 PTX – Two balloons with 6 µg paclitaxel/mm² balloon surface. Significant results are asterisked

| Treatment group | POBA | 1x3 PTX | 1x6 PTX | 2x6 PTX | p-overall | p vs. POBA | | | p vs. 1x3 PTX | | p vs. 1x6 PTX |
| --- | --- | --- | --- | --- | --- | --- | --- | --- | --- | --- | --- |
| Analyzed vessel, n | 8 | 8 | 8 | 8 |  | 1x3 PTX | 1x6 PTX | 2x6 PTX | 1x6 PTX | 2x6 PTX | 2x6 PTX |
| Vessel diameter, mm | 1.60±0.23 | 1.86±0.22 | 1.94±0.41 | 1.82±0.36 | 0.1846 | 0.3673 | 0.1551 | 0.5362 | 0.9524 | 0.9950 | 0.8771 |
| Lumen diameter, mm | 0.83±0.11 | 1.01±0.11 | 1.13±0.25 | 1.08±0.29 | *0.0324 | 0.2930 | *0.0277 | 0.1025 | 0.6351 | 0.9147 | 0.9572 |
| Neointimal diameter, mm | 0.52±0.24 | 0.49±0.08 | 0.49±0.20 | 0.40±0.15 | 0.5869 | 0.9924 | 0.9934 | 0.5658 | >0.9999 | 0.7277 | 0.7199 |
| Medial area, mm² | 1.00 ± 0.28 | 1.34 ± 0.23 | 1.58 ± 0.56 | 1.46 ± 0.60 | 0.0876 | 0.8728 | 0.0988 | 0.3202 | >0.9999 | >0.9999 | >0.9999 |
| External elastic membrane area, mm² | 1.75 ± 0.45 | 2.56 ± 0.53 | 2.87 ± 1.15 | 2.57 ± 0.91 | 0.0577 | *0.0306 | 0.1347 | 0.2306 | >0.9999 | >0.9999 | >0.9999 |
| Lumen area, mm² | 0.53±0.18 | 0.76±0.15 | 1.09±0.46 | 0.89±0.42 | *0.0159 | 0.4842 | *0.0099 | 0.1662 | 0.2181 | 0.8778 | 0.6483 |
| Stenosis area, % | 72.99±7.52 | 71.11±3.51 | 64.54±5.66 | 64.97±9.56 | *0.0445 | 0.9452 | 0.0863 | 0.1292 | 0.2410 | 0.3227 | 0.9994 |
| Inflammation | 2.75±0.46 | 2.88±0.35 | 3.00±0.00 | 2.63±0.52 | 0.2715 | 0.9175 | 0.5808 | 0.9175 | 0.9175 | 0.5808 | 0.2408 |
| Injury | 2.75±0.46 | 2.88±0.35 | 3.00±00 | 2.63±0.52 | 0.2715 | 0.9175 | 0.5808 | 0.9175 | 0.9175 | 0.5808 | 0.2408 |

**Supplementary table 2: Analysis of histomorphometric parameters in the balloon treated segment**

Values are mean±standard deviation or counts n. One-way ANOVA was used to test statistical significance. Bonferroni’s post-hoc test was used for multi comparisons. POBA – Plain old balloon angioplasty. 1x3 PTX – 3 µg paclitaxel/mm² balloon surface. 1x6 PTX – 6 µg paclitaxel/mm² balloon surface. 2x6 PTX – Two balloons with 6 µg paclitaxel/mm² balloon surface**.** Significant results are asterisked.

**Supplementary table 3: Analysis of histomorphometric parameters in the untreated segment**

| Group | POBA | 1x3 PTX | 1x6 PTX | 2x6 PTX | p-overall | p vs. POBA | | | | p vs. 1x3 PTX | | p vs. 1x6 PTX |
| --- | --- | --- | --- | --- | --- | --- | --- | --- | --- | --- | --- | --- |
| Analyzed vessel, n | 8 | 8 | 8 | 8 |  | 1x3 PTX | 1x6 PTX | 2x6 PTX | | 1x6 PTX | 2x6 PTX | 2x6 PTX |
| Vessel diameter, mm] | 1.63±0.22 | 1.66±0.23 | 1.60±0.25 | 1.66±0.26 | 0.9610 | 0.9934 | 0.9958 | | 0.9963 | 0.9625 | >0.9999 | 0.9716 |
| Lumen diameter, mm | 1.15±0.25 | 1.13±0.25 | 1.06±0.12 | 1.13±0.18 | 0.8429 | 0.9956 | 0.8179 | | 0.9933 | 0.9137 | >0.9999 | 0.9255 |
| Neointimal diameter, mm] | 0.30±0.15 | 0.35±0.07 | 0.36±0.15 | 0.34±0.13 | 0.9913 | 0.9982 | 0.9925 | | >0.9999 | 0.9994 | 0.9988 | 0.9936 |
| Lumen area, mm² | 0.86±0.38 | 0.83±0.57 | 0.69±0.15 | 0.86±0.47 | 0.8573 | 0.9989 | 0.8710 | | >0.9999 | 0.9248 | 0.9990 | 0.8734 |
| Stenosis area, % | 55.06±21.67 | 64.86±17.09 | 63.81±7.80 | 60.89±12.71 | 0.9237 | 0.9765 | 0.9953 | | 0.9964 | 0.9983 | 0.9161 | 0.9674 |

Values are mean±standard deviation. One-way ANOVA was used to test statistical significance. Bonferroni’s post-hoc test was used for multi comparisons. POBA – Plain old balloon angioplasty. 1x3 PTX – 3 µg paclitaxel/mm² balloon surface. 1x6 PTX – 6 µg paclitaxel/mm² balloon surface. 2x6 PTX – Two balloons with 6 µg paclitaxel/mm² balloon surface.

**Supplementary table 4: Analysis of histomorphometric parameters in the stent treated segment**

| Group | POBA | 1x3 PTX | 1x6 PTX | 2x6 PTX | p-overall | p vs. POBA | | | | p vs. 1x3 PTX | | p vs. 1x6 PTX |
| --- | --- | --- | --- | --- | --- | --- | --- | --- | --- | --- | --- | --- |
| Analyzed vessel, n | 8 | 8 | 8 | 8 |  | 1x3 PTX | 1x6 PTX | 2x6 PTX | | 1x6 PTX | 2x6 PTX | 2x6 PTX |
| Vessel diameter, mm | 3.31±0.32 | 3.42±0.17 | 3.37±0.27 | 3.42±0.36 | 0.8359 | 0.8566 | 0.9717 | | 0.8595 | 0.9840 | >0.9999 | 0.9829 |
| Lumen diameter, mm | 1.85±0.57 | 2.32±0.50 | 2.75±0.23 | 2.81±0.33 | *0.0005 | 0.1613 | *0.0015 | | *0.0011 | 0.2068 | 0.1432 | 0.9928 |
| Neointimal diameter, mm] | 1.01±0.36 | 0.80±0.42 | 0.29±0.06 | 0.40±0.17 | *0.0001 | 0.5056 | *0.0002 | | *0.0026 | *0.0085 | 0.0670 | 0.8651 |
| Lumen area, mm² | 3.09±1.84 | 4.49±1.68 | 5.87±1.07 | 6.09±1.73 | *0.0034 | 0.3208 | *0.0093 | | *0.0063 | 0.3368 | 0.2413 | 0.9929 |
| Stenosis area, % | 66.15±16.12 | 50.52±17.42 | 36.07±10.01 | 33.66±8.03 | *0.0002 | 0.1256 | *0.0008 | | *0.0005 | 0.1730 | 0.1038 | 0.9861 |
| Inflammation | 2.98±0.04 | 2.97±0.08 | 2.98±0.07 | 3.00±0.00 | 0.7626 | 0.9708 | 0.9846 | | 0.9512 | 0.9997 | 0.7672 | 0.8140 |
| Injury | 2.99±0.04 | 2.97±0.08 | 2.98±0.07 | 3.00±0.00 | 0.7626 | 0.9708 | 0.9846 | | 0.9512 | 0.9997 | 0.7672 | 0.8140 |

Values are mean±standard deviation. One-way ANOVA was used to test statistical significance. Bonferroni’s post-hoc test was used for multi comparisons. POBA – Plain old balloon angioplasty. 1x3 PTX – 3 µg paclitaxel/mm² balloon surface. 1x6 PTX – 6 µg paclitaxel/mm² balloon surface. 2x6 PTX – Two balloons with 6 µg paclitaxel/mm² balloon surface. Significant results are asterisked.

**Supplementary table 5: P-values for comparison of quantitative parameters of coronary innervation**

| Treatment group | | POBA | 1x3 PTX | 1x6 PTX | 2x6 PTX |
| --- | --- | --- | --- | --- | --- |
| S100-staining | | | | | |
| Vessel segment | | untreated vs. balloon  balloon | untreated vs. balloon  balloon | untreated vs. balloon  balloon | untreated vs. balloon  balloon |
| Nerve fiber density, nerves/cm² | | 0.7186 | 0.3779 | **0.0007** | 0.1170 |
| Distance to lumen, mm | | **0.0009** | **0.0005** | **0.0073** | **0.0092** |
| Nerve fiber size, µm² | | 0.6730 | **0.0359** | 0.1451 | 0.1953 |
|  | <500 µm², % | **0.0267** | 0.0680 | **0.0028** | **0.0391** |
|  | 500 - 1000 µm², % | 0.2785 | 0.3163 | 0.3751 | 0.9338 |
|  | 1000 - 5000 µm², % | **0.0320** | **0.0129** | **0.0481** | 0.1020 |
|  | >5000 µm², % | 0.2781 | **0.0107** | 0.1829 | 0.1187 |
| TH-staining | | | | | |
| Nerve fiber density, nerves/cm² | | 0.6439 | **0.0020** | **0.0003** | **0.0478** |
| Distance to lumen, mm | | **0.0021** | **0.0060** | **0.0400** | **0.0487** |
| Nerve fiber size, µm² | | 0.3297 | 0.0814 | **0.0372** | **0.0410** |
|  | <500 µm², % | 0.2250 | **0.0276** | **0.0105** | 0.0581 |
|  | 500 - 1000 µm², % | 0.4600 | 0.4182 | **0.0009** | 0.0883 |
|  | 1000 - 5000 µm², % | 0.1038 | 0.0810 | 0.0529 | 0.1232 |
|  | >5000 µm², % | 0.8104 | 0.0644 | 0.0720 | 0.3048 |

**Supplementary figure 1: Representative images of histological sections used for analyses**


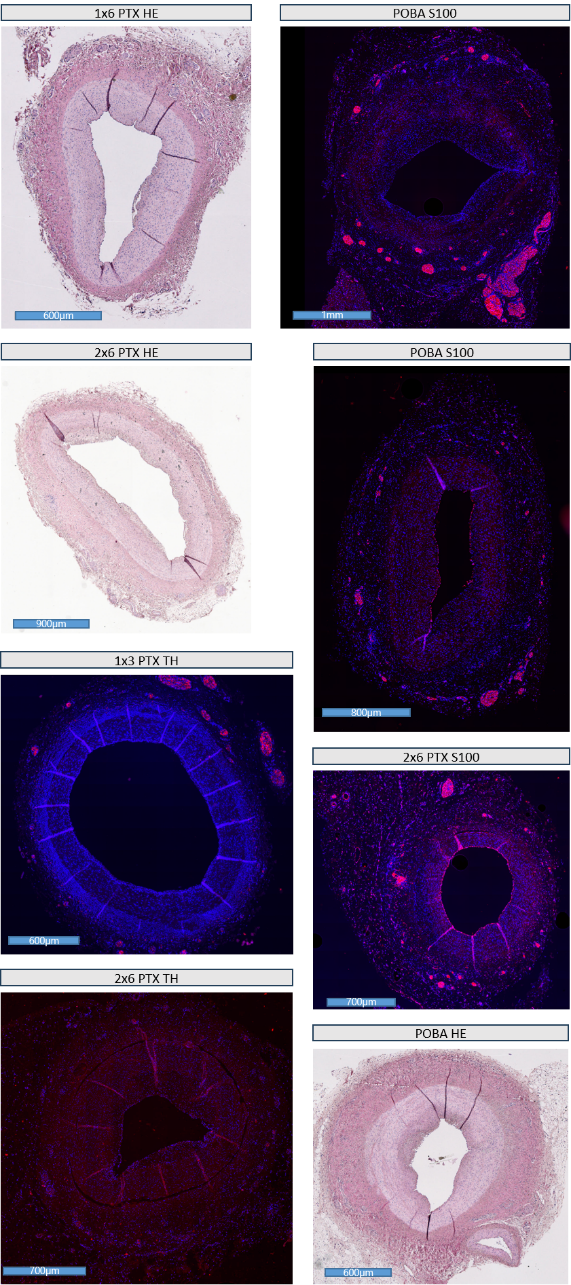


Representative sections from the four treatment groups and the stainings used for the analyses are shown.

**Supplementary figure 2: Lumen diameter, lumen area and neointimal thickness in the stent treated segment**

**
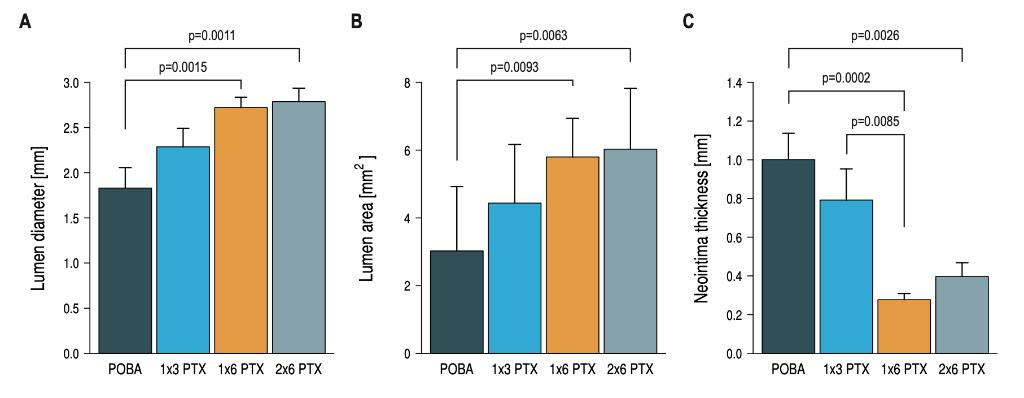
**

Lumen diameter **(A)**, lumen area **(B)**, and neointimal thickness **(C)** in the stent-treated segment between the four treatment groups. Statistical significance of Bonferroni’s post-hoc test for multi comparison between individual groups is indicated by a bracket and the corresponding p-value. Error bars represent the standard deviation.

**Supplementary figure 3: EVG staining of balloon-treated coronary segments**


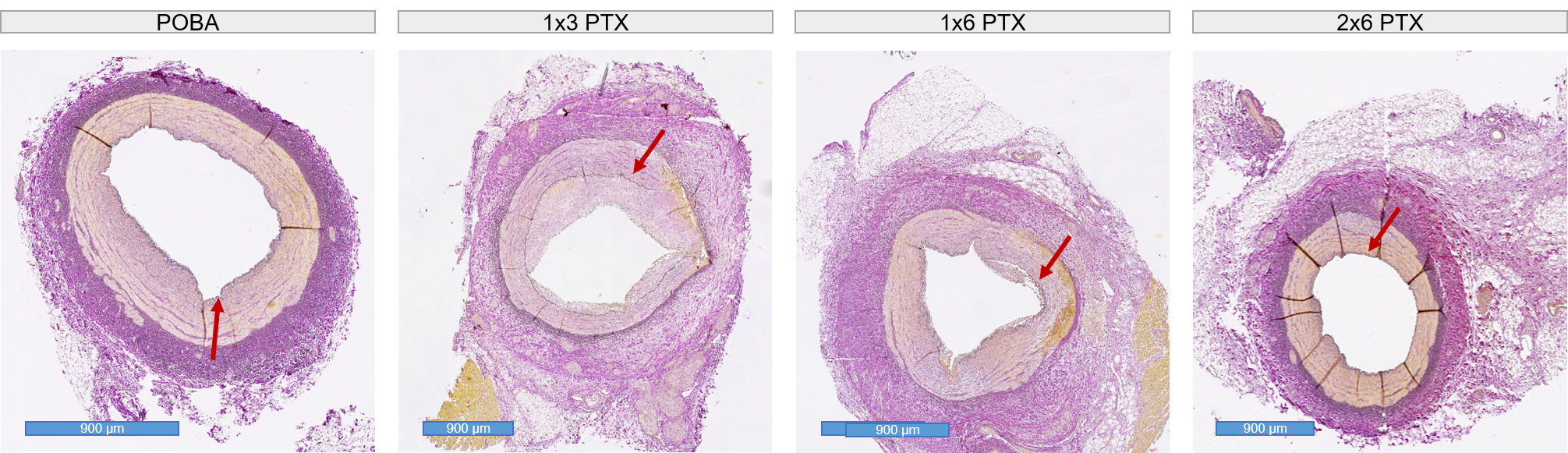


Representative Elastica van Gieson-stained cross-sections from balloon-treated coronary segments in the POBA, 1×3 PTX, 1×6 PTX, and 2×6 PTX groups. EVG staining was performed to evaluate medial architecture and the integrity of the internal elastic lamina (IEL). Across treatment groups, medial architecture remained identifiable despite procedure-related vascular injury. The IEL was mostly continuous, although focal irregularities or limited disruptions were occasionally observed. These changes were not restricted to PTX-treated vessels and did not show a dose-dependent pattern. Red arrows indicate the IEL.

**Supplementary figure 4: Assessment of peri- and endoneural fibrosis across treatment groups**


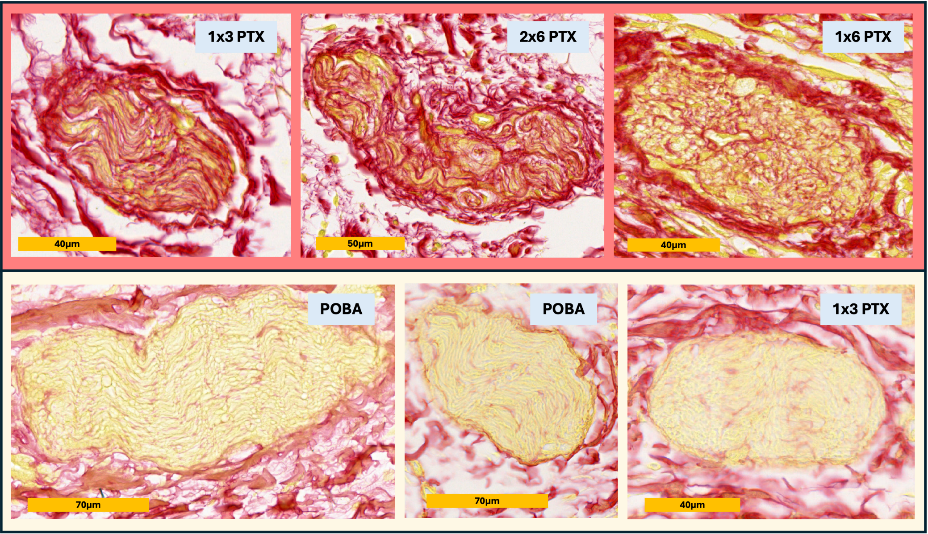


Representative perivascular nerve fibers from the balloon-treated regions of the different groups stained with Sirius Red. PTX-treated groups exhibited pronounced fibrosis (red).

**Supplementary figure 5: Representative images of nerve fibers affected by the procedure**


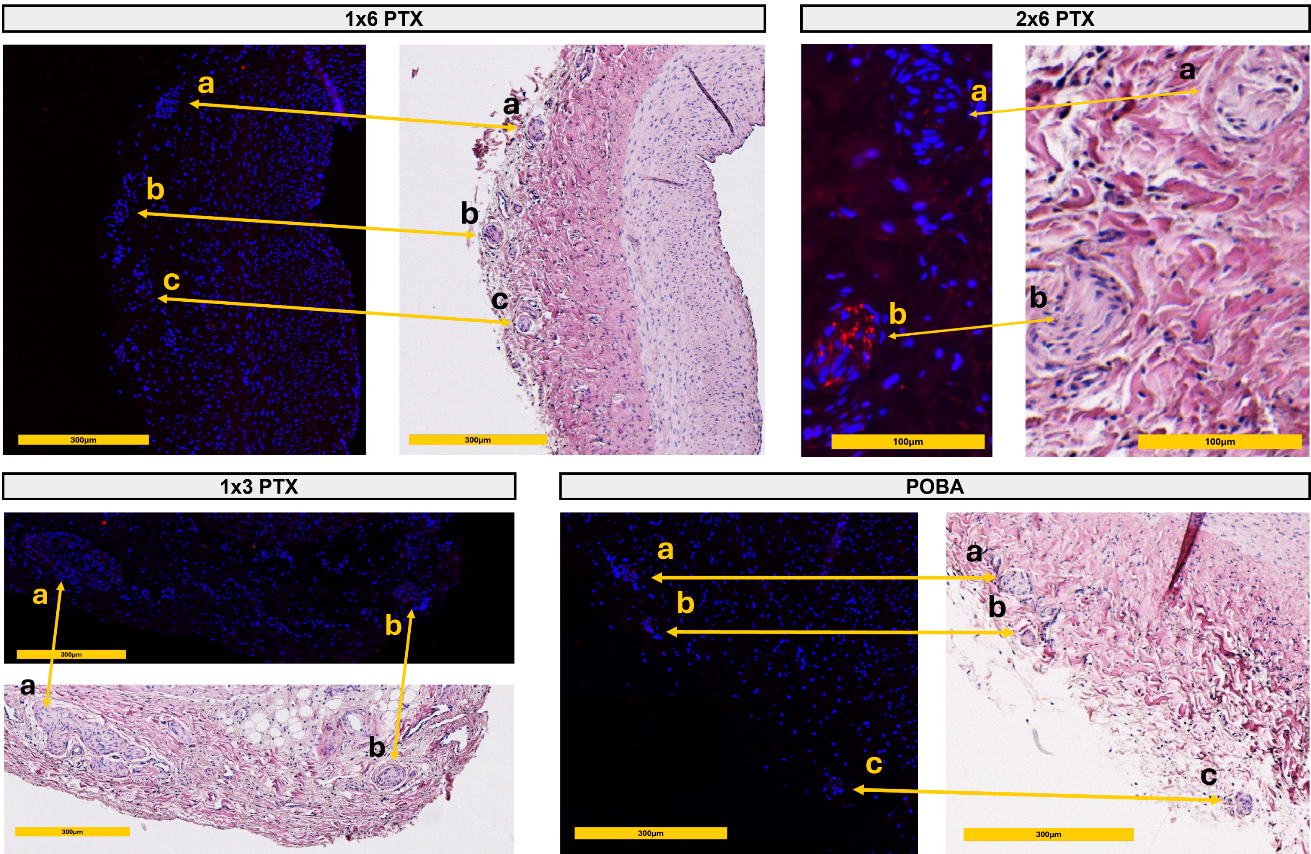


Representative images of balloon treated segments from each group illustrating functional nerve fiber damage. This is characterized by absence of TH immunostaining, while the corresponding fibers remain visible in H&E staining. Yellow arrows indicate the respective nerve fibers, with identical letters marking corresponding fibers across stainings.

**Supplementary figure 6: Mean nerve fiber density in the untreated and balloon treated segments in S100- and TH-staining**

**
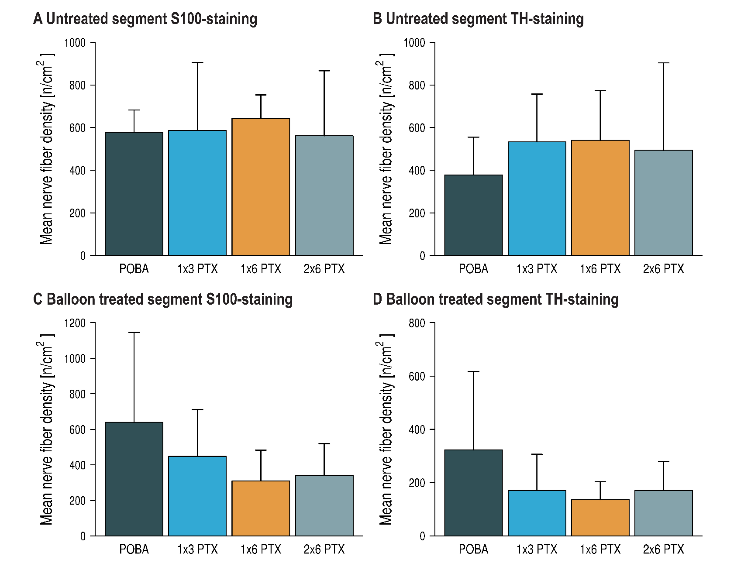
**

Mean nerve fiber density in the untreated **(A+B)** and balloon-treated **(C+D**) segments between the four treatment groups in S100- and TH-staining. Error bars represent the standard deviation.

**Supplementary figure 7: TH/S100 positive nerve fiber ratio**


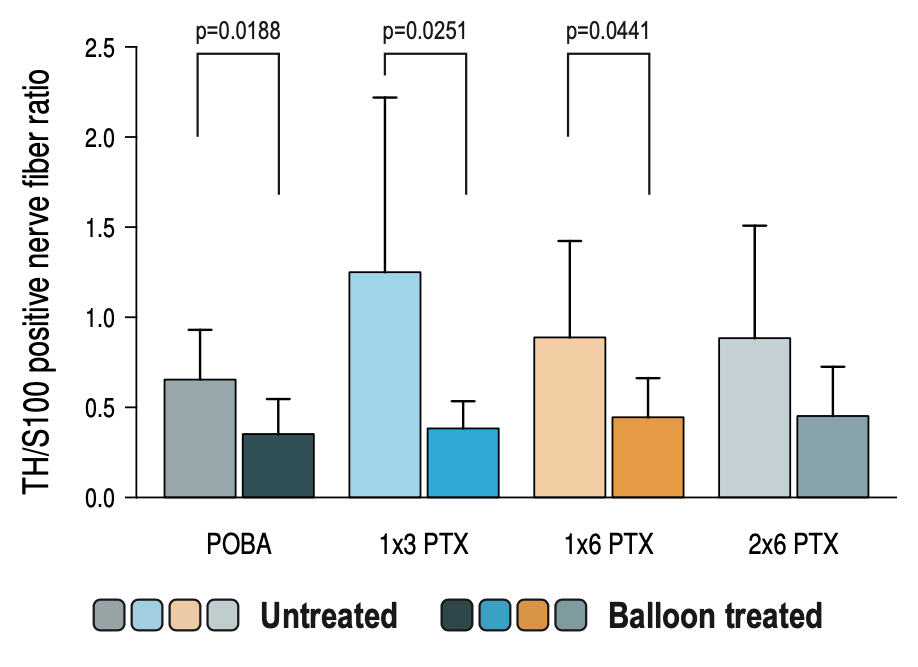


Comparison of TH/S100 positive nerve fiber ratio between the untreated and treated segments in the four treatment groups. Statistical significance of Bonferroni’s post-hoc test for multi comparison between individual groups is indicated by a bracket and the corresponding p-value. Error bars represent the standard deviation.

**Supplementary figure 8: Per-vessel association between TH-positive nerve fiber loss and late lumen change**


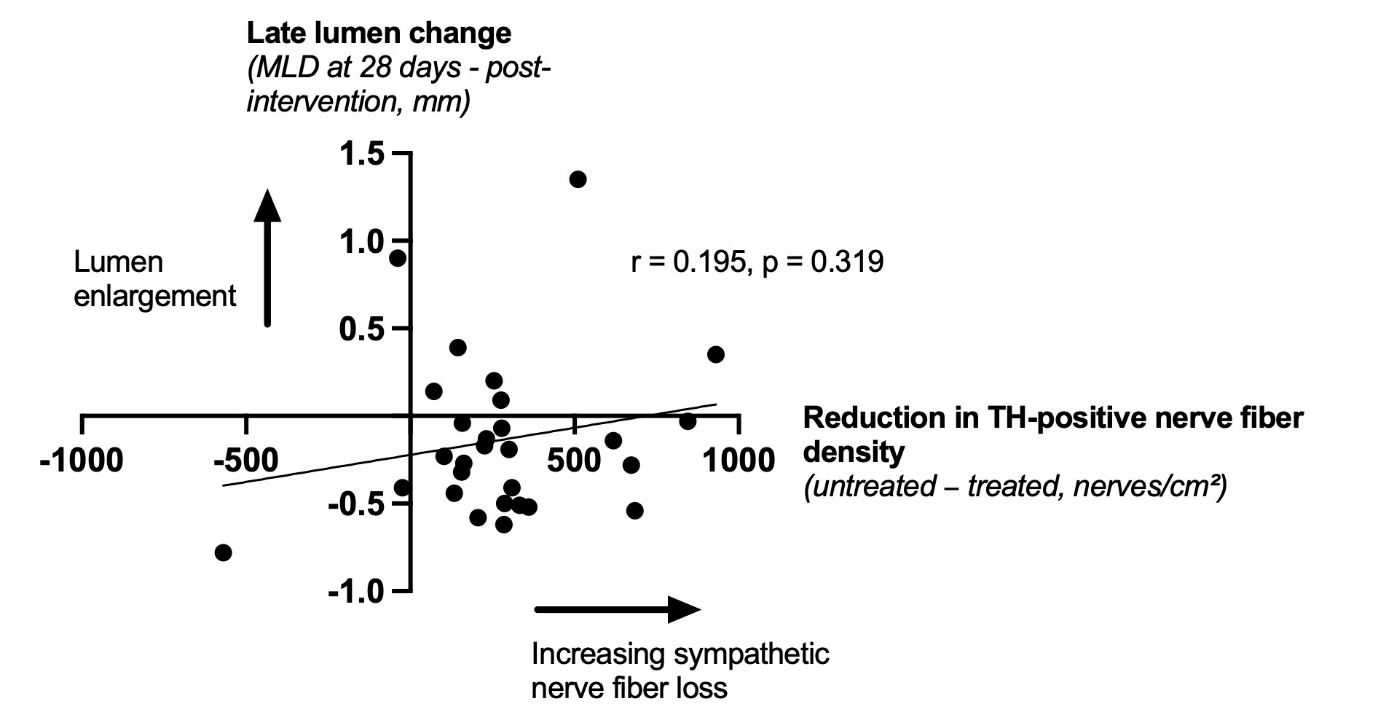


Scatter plot showing the per-vessel relationship between the reduction in TH-positive nerve fiber density and late lumen change in balloon-treated coronary segments. TH-positive nerve fiber loss was calculated as TH-positive nerve fiber density in the untreated reference segment minus TH-positive nerve fiber density in the corresponding balloon-treated segment. Late lumen change was calculated as minimal lumen diameter at 28-day follow-up minus post-interventional minimal lumen diameter. Positive values indicate luminal gain, whereas negative values indicate late lumen loss. Numerically, increasing TH-positive nerve fiber loss tended to be associated with less late lumen loss and more pronounced luminal enlargement. MLD – minimal lumen diameter. TH – tyrosine hydroxylase.
